# New Editors-in-Chief and future directions: a glimpse into the evolving future of Neurogenetics

**DOI:** 10.1007/s10048-023-00740-w

**Published:** 2023-12-29

**Authors:** Geraldine Zimmer-Bensch

**Affiliations:** Division of Neuroepigenetics, RWTH Aachen University, Worringerweg 3, Aachen, 52074 Germany

With the start of a new year we, Prof. Geraldine Zimmer–Bensch and Dr. Silvia De Rubeis, are delighted to introduce ourselves as the new Editors-in-Chief of Neurogenetics. With this and the next editorial, we would like to present ourselves to the readers and inform the community about the coming changes in aims and scope.

We also would like to take this opportunity to thank Prof. Manuel B. Graeber, Prof. Georg W. J. Auburger, and Prof. Louis J. Ptáček for their years of dedication to the journal and their passion for disseminating scientific knowledge.

I am Geraldine Zimmer–Bensch, a Professor for Neuroepigenetics at RWTH Aachen University in Germany. My academic journey began at Friedrich–Schiller University in Jena, Germany, under the guidance of Prof. Dr. Jürgen Bolz, where my fascination with the intricacies of the brain and the formation of its diverse cellular constituents took root.

During my PhD, it became evident that humans only have approximately 20,000 protein-coding genes. For this, I was seeking answers in the proteome and later in the code of proteoglycans during my postdoctoral training in Rio de Janeiro, Brazil, as instructors for brain development.

When I became a principal investigator of the “Neuroepigenetics research group” at the University Hospital Jena, I returned to the realm of genes, particularly focusing on the epigenome as the “second code.” Following my appointment as a Professor for Neuroepigenetics at RWTH Aachen University, I integrated these diverse aspects. My current research explores how “environmental information,” such as proteins expressed in the membrane of neighboring cells, influences the epigenome, and through this gene expression and physiological responses that guide discrete neurodevelopmental processes in cerebral cortex formation.

Furthermore, our investigations delve into understanding how epigenetic mechanisms impact neuronal function, behavior, and brain cancer. We explore the integration of external stimuli into the neuronal genome, leaving molecular traces or even scars that evoke context-dependent, specific physiological responses in both health and disease.

I believe I am not alone with my motivation for taking on editorial duties, rooting in my personal experiences as an author—encountering non-constructive reviews and challenges in publishing data supporting “unconventional” hypotheses, particularly as a young and less established Principal Investigator.

Therefore, my primary goals as an editor are to enhance the fairness, constructiveness, supportiveness, and transparency of the peer review process. I am committed to assisting early-career scientists and PIs, and those exploring “unconventional hypotheses”. I also emphasize the significance of publishing well-designed studies, even when hypotheses are not confirmed, as reporting “negative results” is equally important in my opinion. Upholding best practices and high scientific standards is a key focus. Incorporating the request for raw data, when appropriate, is in my view crucial to address the “reproducibility crisis”.

I am specifically dedicated to increasing the visibility of the journal among neurogenetic scientists involved in basic science, such as those working with animal models, primary neuron cultures, iPSCs, or organoids/assembloids. Additionally, I aim to connect the journal more closely with the neuroepigenetic scientific community in both clinical and basic science.

Overall, my objective is to facilitate effective scientific communication and contribute to maintaining scientific integrity.

I am enthusiastic about collaborating closely with the other new EiC Dr. Silvia De Rubeis, who will introduce herself in the next editorial, our associated editors, and our esteemed Board of Reviewing Editors. Together, we aim to embark on a progressive new chapter in the journal’s successful history, where Neurogenetics will publish findings that contribute to a better understanding of the (epi) genetic basis of normal and abnormal function of the nervous system development and function, as well as of brain dysfunction. In addition to Neurogenetic disorders, studies that address the genetic or epigenetic basis of neurodevelopmental, neuropsychiatric, and neurodegenerative diseases will be the main focus of the journal.

Geraldine Zimmer–Bensch, RWTH Aachen University, Division of Neuroepigenetics

Aachen, Germany,

December 2023

Thank you from the Publisher!

The journal Neurogenetics was founded in 1997 by Professor Ulrich Müller and Professor Manuel Graeber and has been published by Springer–Verlag since 1998. In founding the journal, the two editors demonstrated great foresight and an extraordinary feel for current topics in human genetics research. Neurogenetics quickly established itself in the scientific community as a forum for publishing findings that contribute to a better understanding of the genetic basis of normal and abnormal functions of the nervous system.

Professor Ulrich Müller went into well-deserved retirement at the end of 2017 after 20 years as editor, and Professor Georg Auburger and Professor Louis Ptacek, together with Professor Manuel Graeber, have taken on the responsibility of continuing the journal. The editors have been supported from the beginning and throughout the years by Silke Reichmann, who as Editorial Assistant has tirelessly held the threads together and ensured smooth processes.

The year 2024 now marks a change in the editorial team for Neurogenetics and also in the content focus towards epigenetics topics. We warmly welcome our two new Editors-in-Chief, Professor Geraldine Zimmer–Bensch and Dr. Silvia De Rubeis, and look forward to working with them on the future success of Neurogenetics. Professor Graeber and Ms. Reichmann have kindly agreed to accompany the transition process for another 6 months.

On behalf of Springer Nature, we would like to thank the three long-standing editors and Ms. Reichmann for their excellent cooperation, for their many productive discussions, and for their loyalty and tireless commitment.

Lorena Salgueiro Ferreno, Associate Publisher, Springer Nature

Andrea Pillmann, Executive Publisher, Springer Nature

Heidelberg, Germany

December 2023

