# Correction to: New Editors-in-Chief and future directions: a glimpse into the evolving future of Neurogenetics

**DOI:** 10.1007/s10048-024-00747-x

**Published:** 2024-01-22

**Authors:** Geraldine Zimmer-Bensch

**Affiliations:** Division of Neuroepigenetics, RWTH Aachen University, Worringerweg 3, 52074 Aachen, Germany

**Correction to: Neurogenetics**

10.1007/s10048-023-00740-w

The published online version of the original article contains a mistake.

The name “Professor Claudia Testa” found in the second to the last paragraph should be corrected to “Dr. Silvia De Rubeis”. The sentence should read as “We warmly welcome our two new Editors-in-Chief, Professor Geraldine Zimmer–Bensch and Dr. Silvia De Rubeis, and look forward to working with them on the future success of Neurogenetics.”.

The original article has been corrected.

